# Associations between problem technology use, life stress, and self-esteem among high school students

**DOI:** 10.1186/s12889-024-17963-7

**Published:** 2024-02-16

**Authors:** Blal Idrees, Hugues Sampasa-Kanyinga, Hayley A. Hamilton, Jean-Philippe Chaput

**Affiliations:** 1 Department of Health Sciences, Carleton University, Ottawa, ON Canada; 2 Healthy Active Living and Obesity Research Group, Children’s Hospital of Eastern Ontario Research Institute, 401 Smyth Road, Ottawa, ON K1H 8L1 Canada; 3 Institute for Mental Health Policy Research, Centre for Addiction and Mental Health, Toronto, ON Canada; 4 Dalla Lana School of Public Health, University of Toronto, Toronto, ON Canada; 5 Department of Pediatrics, University of Ottawa, Ottawa, ON Canada

**Keywords:** Screen, Smartphone, Tablet, Computer, Laptop, Problematic internet use, Mental health, Self-worth, Strain, Pressure, Adolescents

## Abstract

**Background:**

Adolescence is a critical period for development, with many risk factors resulting in long-term health consequences, particularly regarding mental health. The purpose of this study was to examine the associations between problem technology use, life stress, and self-esteem in a representative sample of adolescents residing in Ontario, Canada.

**Methods:**

Self-reported data were obtained from a cross-sectional sample of 4,748 students (57.9% females) in grades 9 to 12 (mean age: 15.9 ± 1.3 years) who participated in the 2019 Ontario Student Drug Use and Health Survey. Problem technology use was measured using the 6-item Short Problem Internet Use Test, life stress was assessed using an item from the British Columbia Adolescent Health Survey and self-esteem was assessed using a global measure from the Rosenberg Self-Esteem Scale. Ordinal logistic regression models were adjusted for age, sex, ethnoracial background, subjective socioeconomic status, body mass index z-score, tobacco cigarette smoking, alcohol consumption and cannabis use.

**Results:**

We found that 18.3% of participants reported symptoms of moderate-to-high problem technology use, although symptoms were more common in females than males (22% vs. 14.7%, respectively). Moderate-to-high problem technology use was associated with 2.04 (95% CI: 1.77–2.35) times higher odds of reporting high life stress and 2.08 (95% CI: 1.76–2.45) times higher odds of reporting low self-esteem compared to all other response options.

**Conclusions:**

Findings from this study show that problem technology use is strongly associated with higher life stress and lower self-esteem in adolescents. This study supports the importance of developing and implementing effective strategies that help to mitigate the adverse effects of problem technology use on adolescent mental health.

## Background

Adolescence marks a critical biological, social, and psychological growth and development period. Adverse influences on emotional and psychological well-being can thus impair healthy development within this period of vulnerability [1, 2]. Significant links between self-esteem and life stress have been identified, with both high life stress and low self-esteem during adolescence being strong predictors of poor health and development [1, 3, 4]. These adverse effects include an increased risk of developing mental health disorders such as depression and anxiety [3, 5]. Additionally, problem technology use, which refers to technology usage in a manner which elicits biopsychosocial harm, has also been associated in a systematic review with poor health and developmental outcomes due to adverse effects on sleep and mental health [6].

Mental health among Canadian adolescents has seen a drastic decrease, with only around 40% indicating good or excellent mental health in 2020, compared to 62% in 2018 [7]. Coincidingly, problem technology use among adolescents has continuously increased, with factors such as increasing dependency on technology to manage social and emotional deficits, while self-reported self-esteem levels among adolescents have declined over this period [6, 7]. Additionally, the economic burden of mental illnesses in Canada is significant, with an estimated $51 billion spent annually to cover associated healthcare costs and lost productivity of Canadians [8].

Associations between life stress, self-esteem, and health predictors have previously been investigated, including risk behaviours, sleep quality, and physical activity [2, 6]. However, the associations between problem technology use, life stress, and self-esteem in adolescents are largely unknown. Understanding these relationships will inform future research and provide a framework for investigations on causal pathways. Such information can help inform stakeholders and target the current trends in life stress and self-esteem among adolescents, particularly high school students. Improving these health measures could reduce the prevalence of mental health disorders and thus alleviate some of the associated economic and social burdens.

This study aimed to examine the associations between problem technology use, life stress, and self-esteem in a sample of Canadian high school students. We hypothesized that problem technology use would be associated with higher life stress and lower self-esteem in high school students.

## Methods

### Study design

The sample data were from the 2019 cycle of the biennial Ontario Student Drug Use and Health Survey (OSDUHS). The cross-sectional survey collected data regarding risk behaviours such as gambling, bullying, and mental and physical health from grade 7 to 12 students enrolled in publicly funded schools in Ontario, Canada [9]. The OSDUHS utilizes a two-stage cluster design with a random selection of schools and classes, stratified by region and school type. The survey was designed to be representative of adolescents in Ontario (Canada). Overall, 14,142 students from 263 schools across 47 school boards participated in the 2019 survey, yielding a student participation rate of 59%, which is above average for a survey requiring active parental or guardian consent [10]. About 12% of students did not participate due to absence during the survey collection period, with another 29% due to failure to submit parental permission forms. In addition to parental consent, students under 18 provided signed permission before participation in the study. Ethical approval for the 2019 OSDUHS was provided by the Research Ethics Boards at the Centre for Addiction and Mental Health as well as at York University and 34 individual school board research review committees. Additional information regarding the 2019 OSDUHS can be found in the full report [11].

### Participants

Two survey versions (Form A and Form B) were randomly distributed to students within the classroom. Analyses were restricted to high school students (grades 9–12) who completed Form A of the survey (n = 5,273), which contained questions related to problem technology use and mental health. Thus, analyses were not possible with students in grades 7 and 8 because they were not asked questions about problem technology use. Participants with missing information on the variables included in the analysis were also excluded (n = 525). This left a final sample of 4,748 participants from 163 schools for this analysis. Students with missing data were significantly more likely than those with complete information to be younger, male, and of Black ethnoracial background.

### Measures

Problem technology use was measured using the Short Problem Internet Use Test (SPIUT). This 6-item questionnaire is a validated test adapted from the Compulsive Internet Use Scale [12]. Participants were asked to rank the frequency of occurrence of the following items: “How often do you find that you are staying on electronic devices longer than you intended?”, “How often do you neglect homework because you are spending more time on electronic devices?”, “How often are you criticized by your parents or your friends about how much time you spend on electronic devices?”, “How often do you lose sleep because you use electronic devices late at night?”, “How often do you feel nervous when you are not using electronic devices and feel relieved when you do go back to using them?”, and “How often do you choose to spend more time on electronic devices rather than go out with friends?” Response options included “never”, “rarely”, “from time to time”, “quite often” and “very often” and were ranked from 0 to 4, respectively. Responses were summed for a final score between 0 and 24, with a cut-off score of 14 and above indicating moderate-to-high problem technology use [12]. Internal reliability was assessed using the Cronbach’s alpha coefficient. The coefficient value for the sample data was 0.78, demonstrating high internal reliability.

Life stress was measured using a statement item derived from the British Columbia Adolescent Health Survey [13]. Participants were asked to rank whether they experienced stress, strain, or pressure in the previous four weeks. Response options included “not at all”, “yes, a little”, “yes, some”, “yes, a lot”, and “yes, almost more than I could take.” Response options were coded from 1 to 5, respectively. Higher scores indicated higher life stress.

Self-esteem was measured using a statement item from the Rosenberg Self-Esteem Scale, a global measure of self-esteem for research purposes with strong reliability and validity among adolescents [14, 15]. Participants were asked to rate their level of agreement with the following statements: “On the whole, I am satisfied with myself”. Response options included “strongly agree”, “somewhat agree”, “somewhat disagree” and “strongly disagree”, and were coded from 1 to 4, respectively. Higher score values indicated lower self-rated self-esteem.

Covariates in this analysis included age (years), sex (male or female), ethnoracial background as a proxy for racism-related stress (White, Black, East/South-East Asian, South Asian, Other [including Chinese, Indigenous, Filipino, Latin American, Central American, South American, West Asian or Arab, Korean, Japanese and Not sure]), subjective socioeconomic status, body mass index (BMI) z-scores, tobacco cigarette smoking, alcohol consumption and cannabis use. Subjective socioeconomic status was measured using an adapted version of the MacArthur Scale of Subjective Social Status, a reliable measure of subjective social status [16, 17]. Scores from 1 (worst off) to 10 (best off) are provided and higher scores are indicative of higher socioeconomic status. Mean values were used for analysis. BMI was calculated using self-reported weight (kilograms) and height (meters), then converted to kilograms/meters^2^. BMI values were then converted to z-scores using the World Health Organization reference data [18]. Participants were asked to rate the frequency of substance use over the past 12 months, which included how often they have smoked cigarettes, consumed alcohol (coolers, beer, wine, liquor), or smoked cannabis. Response options ranged from 1 to 10 for tobacco smoking (from never smoked to ≥ 30 cigarettes a day), 1 to 9 for alcohol consumption (from never drank alcohol to almost every day), and 1 to 7 for cannabis use (from never used to ≥ 40 times). Higher scores reflect a greater frequency of use and median values were used for analysis. Covariates were chosen based on availability in the dataset and their known associations with problem technology use and life stress and self-esteem in the literature.

### Statistical analysis

All analyses were conducted using Stata (version 16.1, Stata Corp., College Station, Texas) and accounted for the weighting and complex sampling design employed by the OSDUHS. We used the svy command in Stata to perform variance estimation that accounts for multiple stages of clustered sampling. Characteristics of the sample were summarized using descriptive statistics, including proportion, mean, and median. Sex differences were assessed using a Pearson chi-square adjusted for the survey design and transformed into an *F*-statistic for categorical data and by an univariable linear regression model for continuous data. Two-way interactions between moderate-to-high problem technology use and sex were examined to test whether the association of moderate-to-high problem technology use with high life stress and low self-esteem varies between males and females. Given that these interactions were not significant (p = 0.11 for life stress and p = 0.36 for self-esteem), data for males and females were pooled to increase statistical power. We also tested for other potential interactions between moderate-to-high problem technology use and other covariates (e.g., age, socioeconomic status, and BMI), but none were found and therefore only pooled analyses are reported. Univariable and multivariable ordinal logistic regression analyses were conducted to examine the association of moderate-to-high problem technology use with high life stress and low self-esteem. Ordered logistic regression was deemed appropriate after tests indicated that the assumption of proportional odds was not violated. Covariates included age, sex, ethnoracial background, subjective socioeconomic status, BMI z-score, tobacco cigarette smoking, alcohol consumption, and cannabis use. Odds ratios (OR) and their 95% confidence intervals (CI) for both univariable and multivariable models were reported.

## Results

Table 1 outlines the sample characteristics. Over half of the participants were female (57.9%) and of White ethnoracial background (52%). We observed that 24.6% of participants reported little life stress in the previous four weeks, 26.9% reported some, and 25.3% reported a lot. Additionally, females were more likely than males to report greater life stress. Nearly 45% of participants somewhat agreed with the self-esteem statement, 26.1% strongly agreed, and 19.3% somewhat disagreed. Females were also more likely to report lower self-esteem than males. We found that 18.3% of participants reported symptoms of moderate-to-high problem technology use, with more females (22%) reporting symptoms of problem technology use than males (14.7%).

**Table 1:** Sample characteristics

	Total sample (N = 4,748)	Males (N = 1,999)	Females (N = 2,749)	p-value^a^
Total (%)	100	42.1	57.9	
**Age (years)**				
Mean (SD) (Min: 11; Max: 20)	15.9 (1.3)	15.9 (1.2)	15.8 (1.4)	0.157
**Grades**				
9	23.0	22.1	23.9	0.586
10	24.5	23.9	25.0	
11	23.9	24.2	23.5	
12	28.7	29.9	27.6	
**Ethnoracial background**				
White	52.0	51.1	53.0	0.481
Black	9.3	9.0	9.5	
East/South-East Asian	14.8	16.0	13.6	
South Asian	8.2	8.0	8.4	
Other	15.8	16.0	15.5	
**Subjective socioeconomic status**				
Mean (SD) (Min: 1; Max: 10)	6.9 (1.7)	6.9 (1.6)	6.8 (1.8)	0.115
**BMI z-score**				
Mean (SD)	0.3 (1.1)	0.4 (1.0)	0.3 (1.3)	0.205
**Tobacco cigarette smoking**				
Median (IQR) (Min: 1; Max: 10)	1.0 (7.0)	1.0 (8.0)	1.0 (6.0)	0.182
**Alcohol consumption**				
Median (IQR) (Min: 1; Max: 9)	3.0 (7.0)	3.0 (8.0)	3.0 (7.0)	0.457
**Cannabis use**				
Median (IQR) (Min: 1; Max: 7) Mean (SD)	1.0 (6.0) 1.92 (1.71)	1.0 (6.0) 2.08 (1.79)	1.0 (6.0) 1.79 (1.74)	< 0.001
**Life stress**				
Not at all	11.3	16.3	6.2	< 0.001
A little	24.6	29.7	19.4	
Some	26.9	27.4	26.4	
A lot	25.3	19.0	31.8	
Almost more than I could take	12.0	7.7	16.3	
Median (Min: 1; Max: 5)	3.0	3.0	3.0	
**Self-esteem**				
Strongly agree	26.1	34.6	17.5	< 0.001
Somewhat agree	44.8	43.8	45.8	
Somewhat disagree	19.3	15.1	23.6	
Strongly disagree	9.8	6.5	13.1	
Median (Min: 1; Max: 4)	2.0	2.0	2.0	
**Moderate-to-high problem technology use**				
No	81.7	85.3	78.0	< 0.001
Yes	18.3	14.7	22.0	

Data are shown as weighted column %, unless otherwise indicated. BMI: body mass index; SD: standard deviation; IQR: interquartile range

For subjective socioeconomic status, higher scores reflect higher status

For substance use variables, response options ranged from 1 to 10 for tobacco smoking (from never smoked to ≥ 30 cigarettes a day), 1 to 9 for alcohol consumption (from never drank alcohol to almost every day), and 1 to 7 for cannabis use (from never used to ≥ 40 times). Higher scores reflect a greater frequency of use

^a^p-value of differences between males and females

Table 2 presents the associations between moderate-to-high symptoms of problem technology use and high life stress among participants based on the univariable and multivariable ordinal logistic regression analysis results. After accounting for covariates, results indicate that moderate-to-high problem technology use was associated with 2.04 (95% CI: 1.77–2.35) times higher odds of reporting high life stress (compared to all other response options). Males were at lower odds of reporting high life stress than females (OR: 0.39; 95% CI: 0.34–0.45). Odds of reporting high life stress also varied by age, subjective socioeconomic status, alcohol consumption and ethnoracial background.

**Table 2:** Associations between moderate-to-high symptoms of problem technology use and high life stress among high school students (N = 4,748)

	Model 1		Model 2	
	OR (95% CI)	p-value	OR (95% CI)	p-value
**Moderate-to-high problem technology use**				
No	1		1	
Yes	2.41 (2.09–2.78)	< 0.001	2.04 (1.77–2.35)	< 0.001
Age	1.14 (1.07–1.21)	< 0.001	1.10 (1.03–1.18)	0.004
**Sex**				
Females	1		1	
Males	0.39 (0.34–0.45)	< 0.001	0.39 (0.34–0.45)	< 0.001
**Ethnoracial background**				
White	1		1	
Black	0.68 (0.51–0.90)	0.008	0.64 (0.49–0.82)	0.001
East/South-East Asian	1.19 (0.99–1.42)	0.060	1.33 (1.10–1.60)	0.003
South Asian	1.06 (0.86–1.30)	0.589	1.23 (0.99–1.53)	0.057
Other	1.07 (0.88–1.30)	0.482	1.13 (0.93–1.37)	0.207
Subjective socioeconomic status	0.90 (0.84–0.95)	0.001	0.91 (0.86–0.97)	0.002
BMI z-score	1.03 (0.97–1.11)	0.340	1.06 (1.00-1.13)	0.058
Smoking tobacco cigarette	1.21 (1.07–1.37)	0.003	1.03 (0.86–1.22)	0.770
Alcohol consumption	1.17 (1.11–1.23)	< 0.001	1.11 (1.05–1.18)	0.001
Cannabis use	1.10 (1.06–1.14)	< 0.001	1.04 (0.98–1.11)	0.220

OR: odds ratio; CI: confidence interval; BMI: body mass index

Model 1 is unadjusted; Model 2 is adjusted for age, sex, ethno-racial background, subjective socioeconomic status, BMI z-score, tobacco cigarette smoking, alcohol consumption, and cannabis use

Table 3 presents the associations between moderate-to-high symptoms of problem technology use and low self-esteem among participants based on the univariable and multivariable ordinal logistic regression analysis results. After accounting for covariates, results indicate that moderate-to-high problem technology use was associated with 2.08 (95% CI: 1.76–2.45) times higher odds of reporting low self-esteem (compared to all other response options). Males were at lower odds of reporting low self-esteem than females (OR: 0.41; 95% CI: 0.36–0.48). Furthermore, subjective socioeconomic status, BMI z-score, cannabis use, alcohol consumption and ethnoracial background were significantly associated with low self-esteem.

**Table 3:** Associations between moderate-to-high symptoms of problem technology use and low self-esteem among high school students (N = 4,748)

	Model 1		Model 2	
	OR (95% CI)	p-value	OR (95% CI)	p-value
**Moderate-to-high problem technology use**				
No	1		1	
Yes	2.55 (2.17–2.98)	< 0.001	2.08 (1.76–2.45)	< 0.001
Age	1.09 (1.03–1.14)	0.002	1.02 (0.97–1.08)	0.433
**Sex**				
Females	1		1	
Males	0.44 (0.38–0.51)	< 0.001	0.41 (0.36–0.48)	< 0.001
**Ethnoracial background**				
White	1		1	
Black	0.72 (0.58–0.90)	0.003	0.63 (0.48–0.83)	0.001
East/South-East Asian	1.51 (1.23–1.85)	< 0.001	1.65 (1.36–2.01)	< 0.001
South Asian	1.07 (0.85–1.33)	0.579	1.29 (1.05–1.58)	0.014
Other	0.91 (0.74–1.12)	0.360	0.90 (0.74–1.08)	0.261
Subjective socioeconomic status	0.74 (0.71–0.78)	< 0.001	0.75 (0.72–0.78)	< 0.001
BMI z-score	1.12 (1.04–1.21)	0.004	1.16 (1.08–1.25)	< 0.001
Tobacco cigarette smoking	1.25 (1.14–1.37)	< 0.001	1.07 (0.95–1.19)	0.270
Alcohol consumption	1.15 (1.09–1.20)	0.001	1.10 (1.04–1.16)	0.001
Cannabis use	1.11 (1.07–1.15)	< 0.001	1.06 (1.01–1.11)	0.026

OR: odds ratio; CI: confidence interval; BMI: body mass index

Model 1 is unadjusted; Model 2 is adjusted for age, sex, ethno-racial background, subjective socioeconomic status, BMI z-score, tobacco cigarette smoking, alcohol consumption, and cannabis use

## Discussion

This study aimed to examine the association between problem technology use, life stress, and self-esteem using a large sample of students in grades 9 to 12 from the 2019 OSDUHS. Results showed strong associations between moderate-to-high problem technology use and high life stress and low self-esteem among adolescents in Ontario, Canada, consistent with our hypothesis. The findings illustrate the need to understand further the role of problem technology use on adolescent mental health and the importance of developing and implementing effective strategies to mitigate these effects.

Previous studies analyzing problem technology use have found associations with adverse mental health outcomes in adolescents, like the findings in this study [19–32]. Twenge et al. (2018) found greater time online in a sample of 1.1 million US adolescents to double the likelihood of unhappiness and lower self-esteem compared to lower use levels [20] in their cross-sectional analysis. Additionally, Tandon et al. (2021) found a correlation between higher screen time and mental health difficulties in 1,000 adolescents but found no evidence of sex-related differences in their cross-sectional study [22]. Khan et al. (2022) found prolonged computer use associated with 46% higher odds of school stress in a cross-sectional sample of 191,786 school-age adolescents [23]. However, these studies have investigated the association alongside other factors such as social media use, sleep, physical activity, academic stress, and health-related risk behaviours. To our knowledge, this is the first study to isolate problem technology use, life stress, and self-esteem within the adolescent population.

Several mechanisms could explain the association between problem technology use and adverse mental health outcomes. First, a literature review showed that addictive screen time use results in brain structural changes that alter cognitive control and emotional regulation, decreasing social coping and increasing ADHD-related behaviour [33]. Poor sleep has also been well documented to increase the risk of adverse mental health outcomes, including higher stress levels and lower self-esteem in cross-sectional studies [20, 34, 35]. High levels of technology use have been found to increase the risk of sleep difficulties in adolescents by altering sleep patterns and circadian rhythmicity in cross-sectional studies, which impair academic performance and increase stress [34, 35]. Moreover, greater levels of screen time have been cross-sectionally associated with lower levels of physical activity and, resultingly, greater stress levels [31, 35]. Physical activity has been shown to mediate stress in a cross-sectional study, as adolescents participating in vigorous-intensity physical activity show improved temperature regulation and beneficial effects on brain neurotransmitters, thereby improving sleep, motivation, and positive emotions [31]. Additionally, increased levels of screen time have been cross-sectionally associated with lower levels of in-person social interaction, thus limiting the psychological benefits of these interactions and decreasing overall psychological well-being [20]. Moreover, greater time online has been linked to poor self-perception regarding body image, particularly in females, and has been associated with cyberbullying [24, 28, 36]. Cyberbullying has been shown to have a cyclical relationship with problem technology use, with victims of cyberbullying reporting greater technology use [36].

However, the findings of this study are inconsistent with some previous research, which has found weak or unclear associations between problem technology use and self-esteem [37, 38]. In a literature review of 13 longitudinal and cross-sectional studies conducted by Stiglic & Viner (2019), only one study indicated moderate evidence for the relationship between screen time and self-esteem; another study demonstrated an association with computer screen-time, but not with mobile phone screen time, with two other studies indicating unclear evidence for an association [37]. A systematic review of 33 longitudinal studies by Tang et al. (2021) also found limited evidence for a relationship between screen time and poor mental health outcomes [38]. Six studies in the review were assessed to determine the relationship between screen time and self-esteem, of which four indicated limited to no evidence for an association [38]. Inconsistencies between our findings and previous research can be attributed to differences in population, study design (cross-sectional versus longitudinal), measures, data collection and analysis (i.e., type of questionnaire), as well as a lack of conclusive research due to the novel nature of the topic of problem technology use. Further studies would require more conclusive study designs and methodologies to assess further the relationship between technology use and adverse mental health outcomes in adolescents.

Results found in this study also highlight significant associations between covariates and the outcome measures. For example, older age, female sex, non-white ethnicity, lower socioeconomic status, higher BMI, alcohol consumption and cannabis use were all significantly associated with high life stress and/or low self-esteem. This finding agrees with the literature on the topic [39] and highlights that high life stress and low self-esteem can be influenced by a variety of factors. It is important to approach these factors holistically, recognizing that multiple factors often interact, and interventions should address both the individual and their broader social context. Professional support from counselors, therapists, and a supportive social network can play a crucial role in mitigating these challenges and promoting positive mental health in adolescents.

Strengths of this study include a large, representative sample size of the adolescent population in Ontario, Canada, an adjustment for relevant confounding factors, and the consideration for sex-related differences in measured outcomes. Despite these strengths, there are several limitations in our analysis. Firstly, a cross-sectional study design limits the ability to draw causal relationships between problem technology use, life stress, and self-esteem. It is also not possible to establish temporality in the observed associations. For example, it is possible that life stress could precipitate higher odds of problem technology use through technology use as a coping mechanism. Additionally, measures derived from the 2019 OSDUHS are from self-reported data, thus increasing the potential for data inaccuracy due to social desirability and recall biases. There is also a potential for selection bias, as adolescents not in school, not present for the survey, or with missing data on the measured variables were excluded from the analysis. Residual confounding of the data by unmeasured variables is another limitation that is always present in epidemiology. These include current medication use, diagnosed mental health issues, and overall health status, which could affect the findings. The generalizability of the findings beyond student in Ontario (Canada) is also unknown.

The study also did not evaluate other factors that have been shown to influence the effect of problem technology use on the measured variables. This includes the type of technology being used (i.e., smartphones, computers, television), the number of devices being used, the time at which it was being used (i.e., weekday, weekend, time of day), and the purpose for use (i.e., social or leisure activities, education). Studies have indicated differences in mental health outcomes in adolescents exhibiting problem technology use depending on the type of technology being used as well as the number of devices, with excessive smartphone use and a higher number of devices associated with more significant disturbances to psychological well-being [19, 23, 25, 29, 30]. Technology use before bedtime also results in worse mental health outcomes due to more significant sleep disturbances [34, 35, 40, 41]. Furthermore, the purpose of technology use has been associated with differences in adverse outcomes, with problem technology use for social purposes (i.e., social media) indicating a decrease in self-esteem [19, 21, 24, 26, 30], whereas excessive technology use for academic purposes and online gaming showing greater life stress [23, 32]. Thus, future research regarding the associations between problem technology use, life stress, and self-esteem should consider the influence of type, timing, number, and purpose to evaluate any associations further.

Future studies should utilize longitudinal and experimental study designs, which would allow for the analysis of causal relationships between the measured variables. The data for this study were also collected before the COVID-19 pandemic in March 2020, which significantly altered technology use by adolescents due to the transition of social and academic activities to a digital platform for up to two years. Therefore, a secondary analysis measuring problem technology use, life stress, and self-esteem since the onset of the COVID-19 pandemic to determine significant changes in the associations discussed in this study should be considered.

## Conclusion

The results of this study highlight the strong association between problem technology use, life stress and self-esteem in adolescents. Future studies with stronger study designs would be needed to understand causal relationships. Temporality was also not established in this cross-sectional study and associations may be occurring in the opposite direction or bidirectionally. Additionally, future studies should better characterize problem technology use by considering context and qualitative aspects of use. Policymakers and stakeholders can use this information to inform policies and strategies to mitigate health risks.

## Data Availability

Requests to access the datasets should be directed to the Centre for Addiction and Mental Health at: hayley.hamilton@camh.ca.

## References

[1] Jafflin K, Constanze Pfeiffer, Bergman MM (2018). Effects of self-esteem and stress on self-assessed health: a Swiss study from adolescence to early adulthood. Qual Life Res.

[2] Sampasa-Kanyinga H, Lien A, Hamilton HA, Chaput JP (2022). Canadian 24-h Movement guidelines, life stress, and self-esteem among adolescents. Front Public Health.

[3] Hoare E, Milton K, Foster C, Allender S (2016). The associations between sedentary behaviour and mental health among adolescents: a systematic review. Int J Behav Nutr Phys Activity.

[4] Herbison CE, Allen K, Robinson M, Newnham J, Pennell C (2017). The impact of life stress on adult depression and anxiety is dependent on gender and timing of exposure. Dev Psychopathol.

[5] Choi Y, Choi SH, Yun JY, Lim JA, Kwon Y, Lee HY, Jang JH (2019). The relationship between levels of self-esteem and the development of depression in young adults with mild depressive symptoms. Medicine.

[6] Elhai JD, Dvorak RD, Levine JC, Hall BJ (2017). Problematic smartphone use: a conceptual overview and systematic review of relations with anxiety and depression psychopathology. J Affect Disord.

[7] Findlay L, Arim R. Canadians report lower self-perceived mental health during the COVID-19 pandemic. Retrieved March 12, 2023, from https:www.statcan.gc.ca.

[8] Moroz N, Moroz I, D’Angelo MS (2020). Mental health services in Canada: barriers and cost-effective solutions to increase access. Healthc Manage Forum.

[9] Tara AB, Robert EM, Mann E, Osduhs HAH. Detailed findings from the ontario student drug use and health survey among ontario students. 2020. www.camh.ca.

[10] Courser MW, Shamblen SR, Lavrakas PJ, Collins D, Ditterline P (2009). The impact of active consent procedures on nonresponse and nonresponse error in Youth Survey Data. Eval Rev.

[11] Book A, Elton-Marshall T, Mann RE, Hamilton HA (2020). Drug Use among Ontario Students, 1977–2019: detailed findings from the Ontario Student Drug Use and Health Survey (OSDUHS).

[12] Siciliano V, Bastiani L, Mezzasalma L, Thanki D, Curzio O, Molinaro S (2015). Validation of a new short problematic internet use test in a nationally representative sample of adolescents. Comput Hum Behav.

[13] Smith A, Forsynth K, Poon C, Peled M, Saewyc E. McCreary Centre Society. Balance and connection in BC: The health and well-being of our youth: Results of the 2018 BC adolescent health survey. 2019;1–108. www.mcs.bc.ca.

[14] Rosenberg M, Schooler C, Schoenbach C (1989). Self-esteem and adolescent problems: modeling reciprocal effects. Am Sociol Rev.

[15] Bagley C, Mallick K (2001). Normative data and mental health construct validity for the Rosenberg self-esteem scale in British adolescents. Int J Adolescence Youth.

[16] Goodman E, Huang B, Schafer-Kalkhoff T, Adler NE (2007). Perceived socioeconomic status: a New type of identity which influences adolescents’ Self Rated Health. J Adolesc Health.

[17] Goodman E, Adler NE, Kawachi I, Frazier AL, Huang B, Colditz GA (2001). Adolescents’ perceptions of social status: development and evaluation of a new indicator. Pediatrics.

[18] WHO Anthro and Macros - Version 3.2.2, January 2011| Global Nutrition Cluster. Retrieved March 26., 2023, from https://www.nutritioncluster.net/resources/who-anthro-and macros-version-322-january-2011.

[19] Ricoy MC, Martínez-Carrera S, Martínez-Carrera I (2022). Social Overview of Smartphone Use by teenagers. Int J Environ Res Public Health.

[20] Twenge JM, Martin GN, Campbell WK (2018). Decreases in psychological well-being among American adolescents after 2012 and links to screen time during the rise of smartphone technology. Emotion.

[21] Barthorpe A, Winstone L, Mars B, Moran P (2020). Is social media screen time really associated with poor adolescent mental health? A time use diary study. J Affect Disord.

[22] Tandon PS, Zhou C, Johnson AM, Gonzalez ES, Kroshus E (2021). Association of Children’s physical activity and screen time with Mental Health during the COVID-19 pandemic. JAMA Netw Open.

[23] Khan A, Lee EY, Horwood S (2022). Adolescent screen time: associations with school stress and school satisfaction across 38 countries. Eur J Pediatrics.

[24] Hrafnkelsdottir SM, Brychta RJ, Rognvaldsdottir V, Chen KY, Johannsson E, Guðmundsdottir SL, Arngrimsson SA (2022). Screen time and body image in Icelandic adolescents: sex-specific cross-sectional and longitudinal associations. Int J Environ Res Public Health.

[25] Liu S, Lan Y, Chen B, He G, Jia Y (2022). Smartphone Use Time and total screen time among students aged 10–19 and the effects on academic stress: a large longitudinal cohort study in Shanghai, China. Front Public Health.

[26] Cachón-Zagalaz J, Sanabrias-Moreno D, Sánchez-Zafra M, Zagalaz-Sánchez ML, Lara-Sánchez AJ (2020). Use of the Smartphone and Self-Concept in University Students according to the gender variable. Int J Environ Res Public Health.

[27] Li Y, Wang Z, You W, Liu X (2022). Core self-evaluation, mental health and mobile phone dependence in Chinese high school students: why should we care. Ital J Pediatr.

[28] Bordeleau M, Gilbert JA, Alméras N, Monthuy-Blanc J, Gagnon J, Mathieu MÈ, Drapeau V (2022). Body image and health-related behaviors among fitspirit participants. BMC Public Health.

[29] Mathew P, Krishnan R (2020). Impact of problematic internet use on the self-esteem of adolescents in the selected school, Kerala, India. Arch Psychiatr Nurs.

[30] Twenge JM, Farley E (2021). Not all screen time is created equal: associations with mental health vary by activity and gender. Soc Psychiatry Psychiatr Epidemiol.

[31] Ge Y, Xin S, Luan D, Zou Z, Bai X, Liu M, Gao Q (2020). Independent and combined associations between screen time and physical activity and perceived stress among college students. Addict Behav.

[32] Li L, Abbey C, Wang H, Zhu A, Shao T, Dai D, Jin S, Rozelle S (2022). The Association between Video Game Time and adolescent Mental Health: evidence from Rural China. Int J Environ Res Public Health.

[33] Lissak G (2018). Adverse physiological and psychological effects of screen time on children and adolescents: literature review and case study. Environ Res.

[34] Nursalam N, Octavia M, Tristiana RD, Efendi F (2019). Association between Insomnia and social network site use in Indonesian adolescents. Nurs Forum.

[35] Marciano L, Camerini AL (2021). Recommendations on screen time, sleep and physical activity: associations with academic achievement in Swiss adolescents. Public Health.

[36] Peláez-Fernández MA, Chamizo-Nieto MT, Rey L, Extremera N (2021). How do Cyber victimization and low core self-evaluations interrelate in Predicting Adolescent Problematic Technology Use?. Int J Environ Res Public Health.

[37] Stiglic N, Viner RM (2019). Effects of screentime on the health and well-being of children and adolescents: a systematic review of reviews. BMJ Open.

[38] Tang S, Werner-Seidler A, Torok M, Mackinnon AJ, Christensen H (2021). The relationship between screen time and mental health in young people: a systematic review of longitudinal studies. Clin Psychol Rev.

[39] Jiménez Boraita R, Gargallo Ibort E, Dalmau Torres JM, Arriscado Alsina D. Lifestyle habits, health indicators and sociodemographic factors associated with health-related quality of life and self-esteem in adolescents. Clin Child Psychol Psychiatry 2023 Sep 1:13591045231200661. doi: 10.1177/13591045231200661. Epub ahead of print. 10.1177/13591045231200661 37658652

[40] Thomée S, Härenstam A, Hagberg M (2012). Computer use and stress, sleep disturbances, and symptoms of depression among young adults - a prospective cohort study. BMC Psychiatry.

[41] Shochat T, Flint-Bretler O, Tzischinsky O (2010). Sleep patterns, electronic media exposure and daytime sleep-related behaviours among Israeli adolescents. Acta Paediatr.

